# Development and characterization of flaxseed mucilage and elastin/collagen biofilms

**DOI:** 10.1016/j.heliyon.2024.e35396

**Published:** 2024-07-29

**Authors:** Genet Tewelde Hailu, Fekadu Lemessa, Melakuu Tesfaye Alemea

**Affiliations:** Department of Chemical Engineering, Adama Science and Technology University, Adama, Ethiopia

**Keywords:** Biofilms, Flaxseed mucilage, Elastin/collagen matrix, Mechanical properties, Food packaging

## Abstract

Flaxseed mucilage (FSM)-based biofilms were prepared with varying compositions of the elastin/collagen (ELN/COL) protein matrix. These biofilms were characterized by using fourier transform infrared spectroscopy (FTIR), thermogravimetric analysis (TGA), differential scanning calorimeter (DSC), and X-ray diffraction (XRD). The thickness, water solubility, moisture content, transparency, and mechanical properties of biofilms were investigated. The biofilms were observed to be homogeneous and flexible. The addition of 40 % w/w ELN/COL to the FSM biofilms (FSM-ELN/COL biofilms) enhanced the thickness from 0.127 to 0.142 mm, water solubility from 59.30 to 84.60 % and elongation at break from 91.4 to 188.6 %. However, the reductions in the tensile strength from 6.56 to 4.69 MPa and melting point from 140 °C to 134 °C of the biofilms were observed. The transparency value of 40 % w/w FSM-ELN/COL biofilms increased from 5.42 to 7.19 due to the presence of ELN/COL within the FSM matrix that hinders the light transmission passing through the biofilm. FTIR and XRD tests indicated hydrogen bonding and electrostatic interactions occurred between FSM and ELN/COL, giving rise to a good compatibility of the system. FSM-ELN/COL biofilms fabricated in this work had appropriate mechanical and thermal stability, thus the promising potential to be employed as food packaging and coating.

## Graphical abstract

**Figure:**
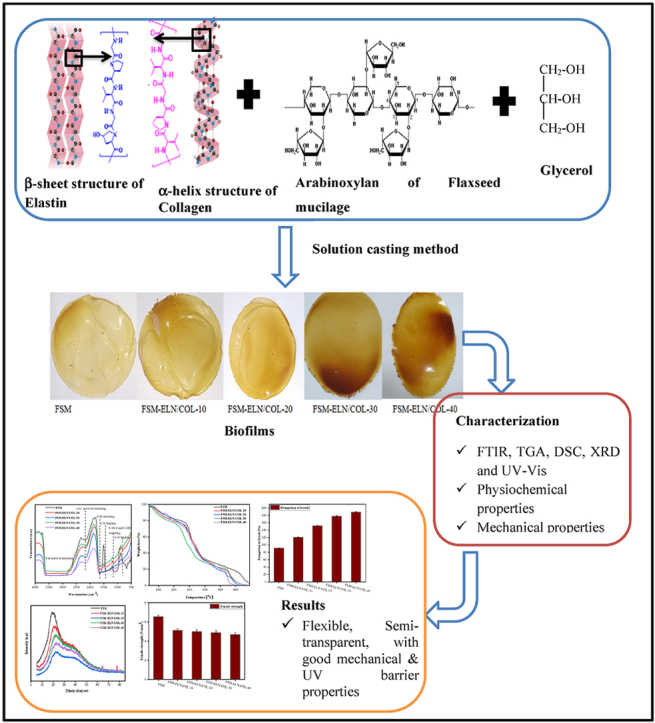

## 1 Introduction

Biopolymer-based film production receives growing interest due to the increasing environmental and food safety concerns caused by petrochemical sources [1,2]. Biopolymers, including polysaccharides, proteins, or lipids derived from natural resources, are highly abundant and easy to recycle after end-use applications such as packaging [3,4]. Hence, many biopolymer films have been used to produce materials for environmentally safe food packaging applications [5]. However, the individual biopolymer films have poor mechanical and barrier properties. Therefore, blending is one encouraging way to overcome the aforementioned limitation by forming chemical interactions through chemical or physical means between biopolymers [[6], [7], [8]]. Many researchers have explored protein/polysaccharide based biofilms such as soy protein isolate/Persian gum, gelatin/cellulose [9], whey protein isolate/cellulose [10], gelatin/chitosan [11], Persian gum/whey protein [12], chitosan/whey protein [13], keratin/starch [14], and PVA/flaxseed mucilage [15].

Flaxseed mucilage (FSM) is one of the natural polymers derived from the whole seeds, meals, and hulls of flaxseed that can be removed by mixing with water under stirring [16]. FSM is a type of polysaccharide with a high content of rhamnose, glucose, galactose, and xylose that provides more sites for physical or chemical modification [17]. FSM has been used as a sustainable alternative for food coating and packaging due to its film-forming ability, low production cost, and easy extraction [18,19]. However, owing to its high hydration rates and inadequate mechanical properties, the use of FSM as an edible film base is limited [19]. One of the strategies to overcome these limitations is to modify and improve the physicochemical properties of FSM films by creating chemical interactions with other biopolymers [15,[20], [21], [22], [23]].

The Elastin/Collagen (ELN/COL) matrix is one of the major components of the extracellular matrix, providing structural stability, strength, and flexibility [24,25]. Therefore, the protein matrix is used for the development of biomaterials with distinctive properties [26]. In this study, the Elastin/Collagen (ELN/COL) matrix was selected as a film-forming biopolymer that might enhance the physicochemical properties of FSM. Therefore, in the present study, biofilms of flaxseed mucilage (FSM) and elastin/collagen matrix (ELN/COL) were prepared for the development of edible films. The biofilms were evaluated for their transparency, thickness, water solubility, and mechanical properties. The biofilms were characterized by Attenuated Total Reflectance Fourier Transform Infrared spectroscopy (ATR-FTIR), thermogravimetric analysis (TGA), differential scanning calorimeter (DSC), X-ray diffraction (XRD), and optical microscope (OM).

## 2 Materials and methods

### 2.1 Materials and reagents

Analytical grade sodium chloride, sodium hydroxide, glycerol and acetone were purchased from Merck. Flaxseed (*Linum usitatissimum* L.*)* and broiler skin were obtained from the local market in Adama City, Ethiopia.

### 2.2 Methods

**Fig. 1:**
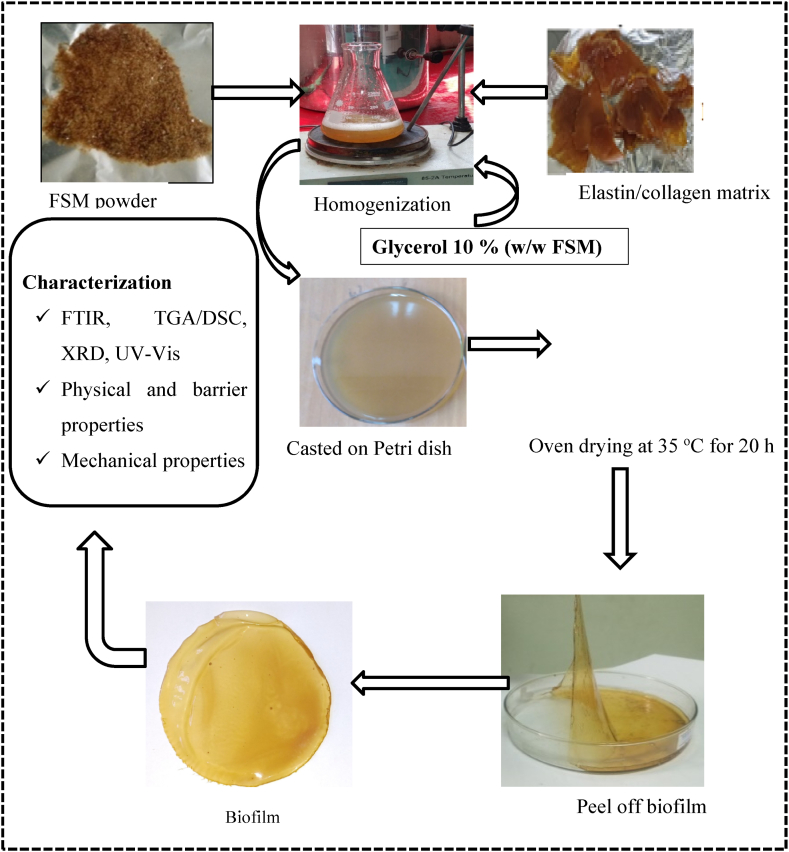
The main steps of the methodology followed for flaxseed mucilage (FSM) and FSM-ELN/COL biofilms development and characterization.

In this section, the details of the extraction protocols followed for flaxseed mucilage and protein matrix (ELN/COL matrix) and biofilm preparation procedures were described and summarized as shown in Fig. 1.

#### 2.2.1 Extraction of flaxseed mucilage

The flaxseed mucilage was obtained by the hot water extraction method. The flaxseeds were washed using distilled water to remove the dust. The samples were added to distilled water and stirred (1:15 w/v) at 90 °C for 3 h on a magnetic hot plate. The resulting solution was filtered with a muslin cloth and dried in an air circulation oven for 24 h at 50 °C to obtain flaxseed mucilage powder.

#### 2.2.2 Extraction of ELN/COL matrix

The extraction of the elastin/collagen matrix from broiler skin was done by the alkaline extraction method as described by Nadalian et al. [27]. Broiler's skins were homogenized in a 1 M NaCl solution for 24 h to remove impurities and then defatted using acetone. The skin was agitated in 0.1 N NaOH solutions and heated for 1 h using a water bath with constant shaking. The fat content was removed by separating funnel and finally the filtrate was dried at 80 °C in the oven to get elastin/collagen.The supporting information table (W1) indicates the proximate analysis of mucilage and protein.

#### 2.2.3 Development of FSM-ELN/COL biofilms

FSM-based biofilms were prepared via the solvent casting method. Firstly, a FSM solution (2 %, w/v) was prepared by dispersing flaxseed mucilage powder in distilled water at 45 °C for 60 min under magnetic stirring. Subsequently, different weights of ELN/COL matrix were added to the FSM solution with mechanical stirring to obtain the mixed solutions with different ratios of FSM to ELN/COL matrix as listed in Table 1. Then glycerol was added as a plasticizer. A film-forming solution was poured on Petri dishes and allowed to dry at 35 °C in a conventional oven for 20 h. Finally, the biofilms were peeled from the Petri dishes and stored in desiccators over silica gel for further use.

**Table 1:** Sample name and composition of FSM-ELN/COL biofilms.

Sample Name	Biofilm composition (w/w)		
	Flaxseed mucilage	Elastin/collagen	Glycerol
FSM	2	–	0.1
FSM-ELN/COL-10	2	0.2	0.1
FSM-ELN/COL-20	2	0.4	0.1
FSM-ELN/COL-30	2	0.6	0.1
FSM-ELN/COL-40	2	0.8	0.1

### 2.3 Characterization of biofilms

#### 2.3.1 Thickness

The thickness of the biofilm samples was measured with a digital hand-held micrometer (Mitutoyo Corporation, Japan) at five different points, and mean values were estimated in mm.

#### 2.3.2 Transparency

A UV–Vis spectrum for biofilms was recorded from 200 to 800 nm wavelength with a UV–Vis spectrophotometer. The transparency value at A600 was obtained using Equation (1).

Equation (1) $$Transparency\;value=\frac{A600}{x}$$

where A600 is the absorbance at 600 nm and x is the biofilm thickness (mm).

#### 2.3.3 Moisture content (MC)

For moisture content analysis, the biofilms were cut into square shapes and subsequently dried at 105 °C for 24 h in an oven. The MC of biofilms was calculated using Equation (2).

Equation (2) $$MC\left(\%\right)=\frac{Mi-Mf\;}{Mi}x\;100$$

where, Mi and Mf are the masses of initial and dried samples, respectively.

#### 2.3.4 Water solubility (WS)

First, the biofilm samples were dried at 105 °C for 24 h to measure their initial weight, and they, were soaked in distilled water at 25 °C for 24 h. After drying, the weight of the undissolved biofilm samples was measured again. The WS biofilm was calculated using Equation (3):

Equation (3) $$WS\left(\%\right)=\frac{Wi-Wf\;}{Wi}x\;100$$

where, Wi and Wf are the initial and final weights of the dried samples, respectively.

#### 2.3.5 Water activity (a_w_)

Water activity of biofilms were recorded using an Aqua Lab Lite water activity meter (Decagon Devices Inc., Pullman, USA), operating at 25 °C.

#### 2.3.6 Mechanical properties

The mechanical properties of the biofilms were determined by using the standard method (ASTM D882-02) with a texture analyzer (Model: TA1, LLOYED). Biofilms were cut in the form of strips with dimensions of 3 × 12 cm. Then the maximum force (N) and deformation (%) were recorded. The tensile strength and Percentage t of elongation were calculated using Equations Equation (4), Equation (5) respectively.

Equation (4) $$Tensile\;strength\;\left(MPa\right)=\frac{\text{maximum}\;\text{load}\;\left(F\right)\;}{\text{cross}\;\text{sectional}\;\text{area}\;\text{of}\;\text{sample}\left(A\right)\;}$$

Equation (5) $$percent\;of\;Elongation\;\left(\%\right)=\frac{b-a\;}{a}\times 100$$

where, (b) final length after being pulled and (a) initial length of the films before being pulled.

#### 2.3.7 FTIR analysis

FTIR spectra of FSM, ELN/COL and biofilms were recorded in the range between 400 and 4000 cm^−1^ wavenumber with a FT/IR-6600typeA using 16 scans at 4 cm^−1^ resolutions.

#### 2.3.8 Thermo-gravimetric analysis (TGA)

Thermogravimetric (TGA) analyses of FSM, ELN/COL and biofilms were carried out under a nitrogen atmosphere using a DTG-60H model (Shimadzu) with a heating rate of 10 °C·min^−1^ from 26 to 700 °C. The weight loss and the different degradation phases of the samples were recorded.

#### 2.3.9 X-ray diffraction (XRD)

X-ray diffraction (XRD) patterns of the biofilms were recorded by a Shizmadzu X-ray diffractometer-7000 in the 2θ range from 5° to 85° with scanning rate of 3°/min.

#### 2.3.10 Differential scanning calorimeter (DSC)

The thermal behaviour of biofilms was analyzed by using a differential scanning calorimeter at a rate of 10 °C/min for the temperature range of 40–200 °C performed under a nitrogen atmosphere.

#### 2.3.11 Optical microscope

The dispersion quality and degree of self-aggregation of biofilms were investigated using an optical microscope using a LV-100 Nikon Eclipse equipped with a Nikon slight camera at 50× magnification.

### 2.4 Statistical analysis

The results were statistically evaluated using analysis of variance (ANOVA) and Tukey's test to compare the means (*p* < 0.05).

## 3 Results and discussion

### 3.1 Percentage yield of FSM

The A. manihot, A. spinosus and T. triangulare yielded yellowish brown, gray and brown amorphous mucilage powders (Fig. 1), respectively. The A. manihot, A. spinosus and T. triangulare yielded yellowish brown, gray and brown amorphous mucilage powders (Fig. 1), respectively. The A. manihot, A. spinosus and T. triangulare yielded yellowish brown, gray and brown amorphous mucilage powders (Fig. 1), respectively.

The hot water extraction method is a very simple, safe and environmentally friendly extraction method that eliminates the use of organic solvents, which are commonly used to precipitate the extract. The flaxseed mucilage yielded yellowish brown mucilage powder with a percentage yield of 6.7 ± 0.3, which is higher than reported by Tulain et al. [28] and lower than the reported values by de Paiva et al. [15] and Hadad and Goli [29] yield reported as 8.5 % and 9.3 %, respectively.

### 3.2 Characterization of flaxseed mucilage and elastin/collagen matrix

#### 3.2.1 Functional group analysis of flaxseed mucilage and elastin/collagen matrix

**Fig. 2:**
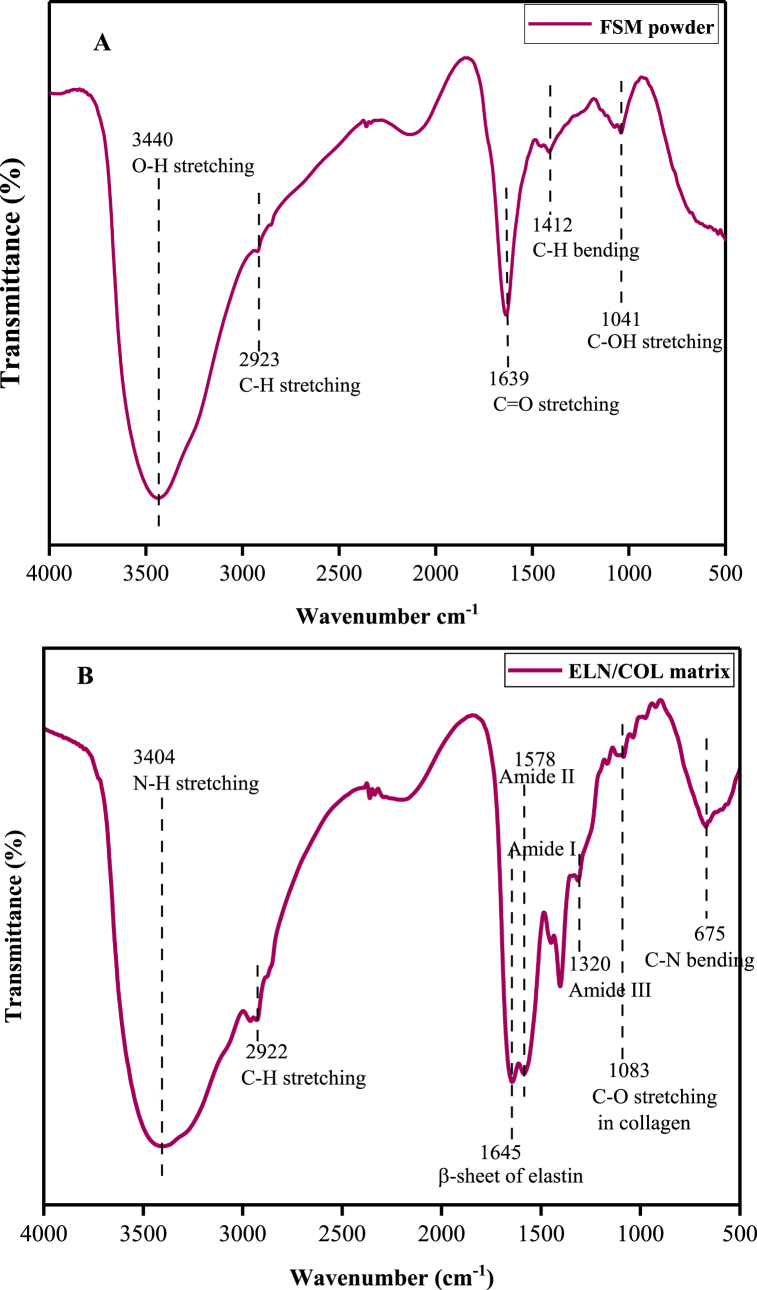
FTIR spectra's of A) flaxseed mucilage powder and B) elastin/collagen matrix.

Fig. 2A presents the FTIR spectrum of flaxseed mucilage (FSM) powder. A broad and intense peak at 3440 cm^−1^ is due to O–H stretching and at 2923 cm^−1^ it is due to CH stretching of sugar [30,31]. The characteristic absorption of the FSM is the band at 1639 cm^−1^, which is ascribed to the stretching vibration of C=O of uronic acids [17,32] and 1412 cm^−1^ assigned to the vibration of C–OH [33,34]. The peak around 1041 cm^−1^ corresponds to the saccharide structure of FSM [35]. The obtained result confirms previous observations [36,37].

The primary characteristic bands for ELN/COL include 3404 cm^−1^ due to N–H stretching and 2922 cm^−1^ due to C–H stretching, as presented in Fig. 2B. The band at 1645 cm^−1^ is due to the C=O stretching vibration of amide I that indicates the β-sheet conformation of elastin [[38], [39], [40]]. The amide II band at 1578 cm^−1^ is due to the presence of N–H plane band and the C–H stretch vibration [24]. The peak of 1320 cm^−1^ is related to amide III and arises from C–OH stretching mode. The peaks at 1452 and 1401 cm^−1^ correspond to bending and wagging vibrations of the side groups of the amino acid structure of ELN/COL [41,42]. The peak at 1083 cm^−1^ indicates the C–O stretching vibration of collagen [43]. Hence, the appearance of peaks at 1645 and 1083 cm^−1^, which allows us to identify the structure of elastin and collagen, implies the coexistence of elastin and collagen in the extracted protein matrix.

#### 3.2.2 Thermal stability of flaxseed mucilage and elastin/collagen matrix

**Fig. 3:**
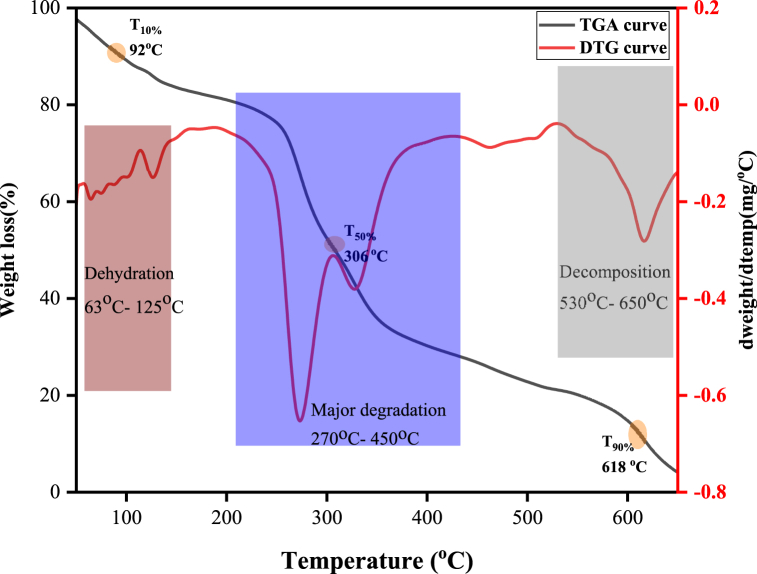
TGA and DTG curves of flaxseed mucilage.

As presented in Fig. 3, pure FSM showed three different weight losses during thermal degradation. The first stage occurred in the temperature range of 46–123 °C, which was attributed to dehydration [44]. The second stage occurred in the temperature range of 182–506 °C (60.16 %), which was attributed to the thermal degradation of FSM [15]. Hadad & Goli [29], also reported 59.3 % weight loss between the temperature ranges of 182–506 °C. The final weight loss stage 540–610 °C (8.88 %) might be caused by the depolymerization and decomposition of the FSM chain [15]. From the DTG curve, the maximum thermal degradation of FSM powder occurred at 330 °C with a weight loss of 23 %.

**Fig. 4:**
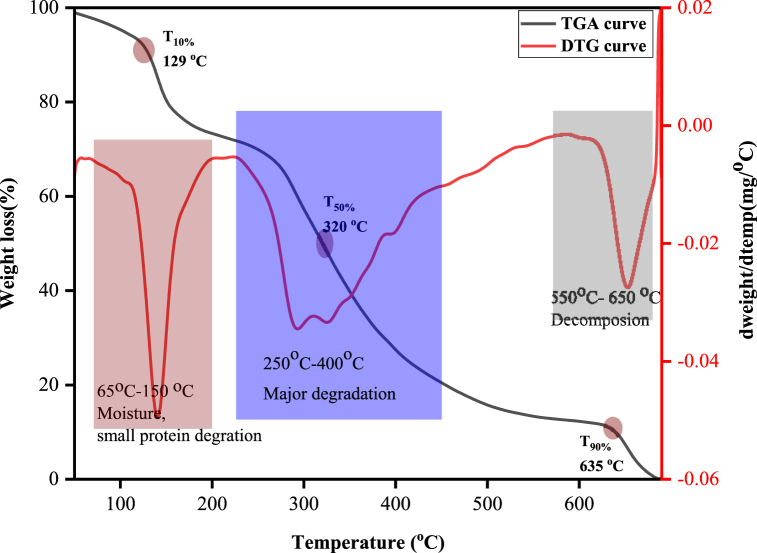
TGA and DTG curves of elastin/collagen matrix.

Fig. 4 designates a total of five stages of mass loss for the extracted ELN/COL matrix. The first peak indicated around 140 °C indicates the evaporation of water with 14 % mass loss. The maximum thermal decomposition occurred in the second stage, around 260–291 °C, which corresponds to the loss of small molecular weight protein fractions with a maximum weight loss of 25 %. The thermal decomposition of high molecular weight proteins occurred beyond 291 °C. The final stage was observed around 652 °C, which is attributed to complete oxidation with a weight loss of 14 %. The T10, T50, Tmax, and T90 of ELN/COL were 129 °C, 320 °C, 291 °C, and 635 °C respectively. The highest degradation rate of the elastin/collagen matrix was recorded in the temperature range of 240–392 °C with a mass loss of 41.34 %. However, M.E. Kibret et al. [45] reported that 30 % weight loss occurs in temperature ranges of 217–446 °C. In another study, the main elastin degradation was achieved in the temperature range of 298–367 °C with a mass loss of 30 % [46].

### 3.3 Characterization of biofilms

#### 3.3.1 Visual appearance of biofilms

**Fig. 5:**
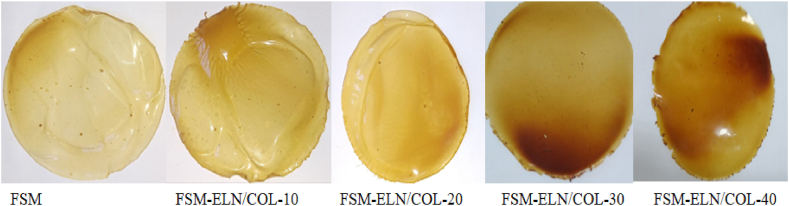
Visual appearance of the flaxseed mucilage based biofilms with 0, 10, 20, 30 and 40 % of ELN/COL matrix.

Fig. 5 show the visual characterization revealed that FSM-based biofilms incorporated with different concentrations of ELN/COL matrix had homogeneous surface with good flexibility and handling ability.

#### 3.3.2 Thickness and water activity

The biofilm thickness increased from 0.127 ± 0.05 to 0.139 ± 0.04 mm, with the increment of elastin/collagen from 0 to 40 % w/w, as shown in Table 2. The FSM-ELN/COL biofilms exhibited slightly higher thickness than the FSM biofilm. The gradual increase in thickness with increasing ELN/COL amount may be attributed to the conformational changes of flaxseed mucilage chains by the addition of ELN/COL and higher solid content. Moreover, this could be attributed to the penetration of the glycerol molecules between polymer chains of the biofilms, which can possibly disrupt molecular interactions between the functional groups of polymer chains, leading to the absorption of more water vapour into the biofilm matrix and increasing the biofilm thickness [47,48]. The result obtained had fulfilled the standard of JIS 1975, which stated that the maximum thickness for edible film is 0.25 mm. The thickness of wheat proteins and alginate films produced by S. Bishnoi et al. [49] ranged from 0.048 to 0.162 mm, when they used wheat proteins ranging from 0 % to 8 % w/v. Moreover, the thickness of casein/chitosan edible film is 0.076–0.087 mm [50]. The water activity of FSM-ELN/COL biofilms ranged from 0.275 to 0.409 as presented in Table 2. These biofilms are microbiologically stable due to the water activity values being below 0.60 [51]. This indicates that there is no microbial growth below this value, so that the biofilms are applicable as packaging material.

**Table 2:** Thickness, water activity (a_w_), moisture content, water in solubility and transparency values obtained from flaxseed mucilage based biofilms.

Films	Thickness (mm)	Water activity (a_w_)	Moisture Content (%)	Water solubility (%)	Transparency value
FSM	0.127 ± 0.05	0.392 ± 0.004	18.88 ± 0.25	59.3 ± 0.16	5.42 ± 0.1
FSM-ELN/COL-10	0.131 ± 0.02	0.275 ± 0.001	21.35 ± 0.18	64.72 ± 0.27	5.75 ± 0.3
FSM-ELN/COL-20	0.135 ± 0.01	0.281 ± 0.002	23.05 ± 0.35	83.92 ± 0.33	6.54 ± 0.2
FSM-ELN/COL-30	0.139 ± 0.03	0.407 ± 0.002	24.49 ± 0.48	84.21 ± 0.19	7.12 ± 0.1
FSM-ELN/COL-40	0.142 ± 0.04	0.409 ± 0.006	24.80 ± 0.23	84.60 ± 0.07	7.19 ± 0.2

#### 3.3.3 Moisture content and water solubility

The moisture content of the biofilms ranged from 18.88 to 24.80 % (Table 2) with a significant influence of ELN/COL. It is possible to observe this same behaviour on pectin/alginate/whey protein concentrate films, in which they showed significant influence of whey protein concentration and linear interactions [52]. The observed moisture content for the biofilm samples can be directly linked to the significant water-holding capacity of flaxseed mucilage and protein matrices. In addition to that, the obtained high moisture content for blended biofilms of FSM and ELN/COL matrix may be due to the weak interactions formed between protein and polysaccharides. These strengthen the interactions between biopolymer components and water molecules. However, the moisture content values for all the biofilms were observed to be lower than those presented for flaxseed mucilage films reported in literature [18,19]. The moisture content of the biofilms undergoes a slight increase due to the attachment of water molecules by hydrogen bonds as provided by carboxyl and hydroxyl groups. Similar results were found for gelatin, tragacanth gum and persian gum based edible biofilms [53] and quinoa protein/hsian-tsao gum biofilms [54].

The water solubility of the FSM-ELN/COL biofilms (Table 2) was measured to be 59.30–84.60 %, which might have been due to the hydrophilic polysaccharide structure of FSM and the moisture sorption and water-binding properties of the N–H groups and carboxyl groups of the ELN/COL matrix [55]. Thus, the attracted water into the polymer matrix, and the hydrophilicity increased. The high moisture content of the biofilms can represent high water solubility. The addition of ELN/COL caused a rise in solubility that reflects the rise of the total unoccupied volume by water molecules. This might be due to the reduction of intermolecular interaction between the polymer chains. Similarly, Wang et al. [56] also reported that the addition of collagen to corn-starch biofilm with the highest content had the highest water solubility.

#### 3.3.4 Transparency

The transparency of the fabricated biofilms was obtained from UV–Visible spectra by taking the absorbance at 600 nm (Table 2). Among all the prepared biofilms, FSM biofilms show the least opaque and higher transparency than the others. The addition of ELN/COL into FSM biofilms reduces the transparency. This phenomenon may be caused by increased diffuse reflection of light as it scatters through the biofilms. The transparency value at 600 nm was found to be 5.42 for FSM control biofilm, while it increased from 5.75 to 7.19 as ELN/COL content increased. The gradual decrease in transparency upon the addition of elastin/collagen protein to the flaxseed mucilage matrix could be the result of interactions between components and network re-arrangements [49]. Moreover, ELN/COL is attributed to the increase in the percentage of biofilm solids that causes low transparency [57].

#### 3.3.5 Mechanical properties

**Fig. 6:**
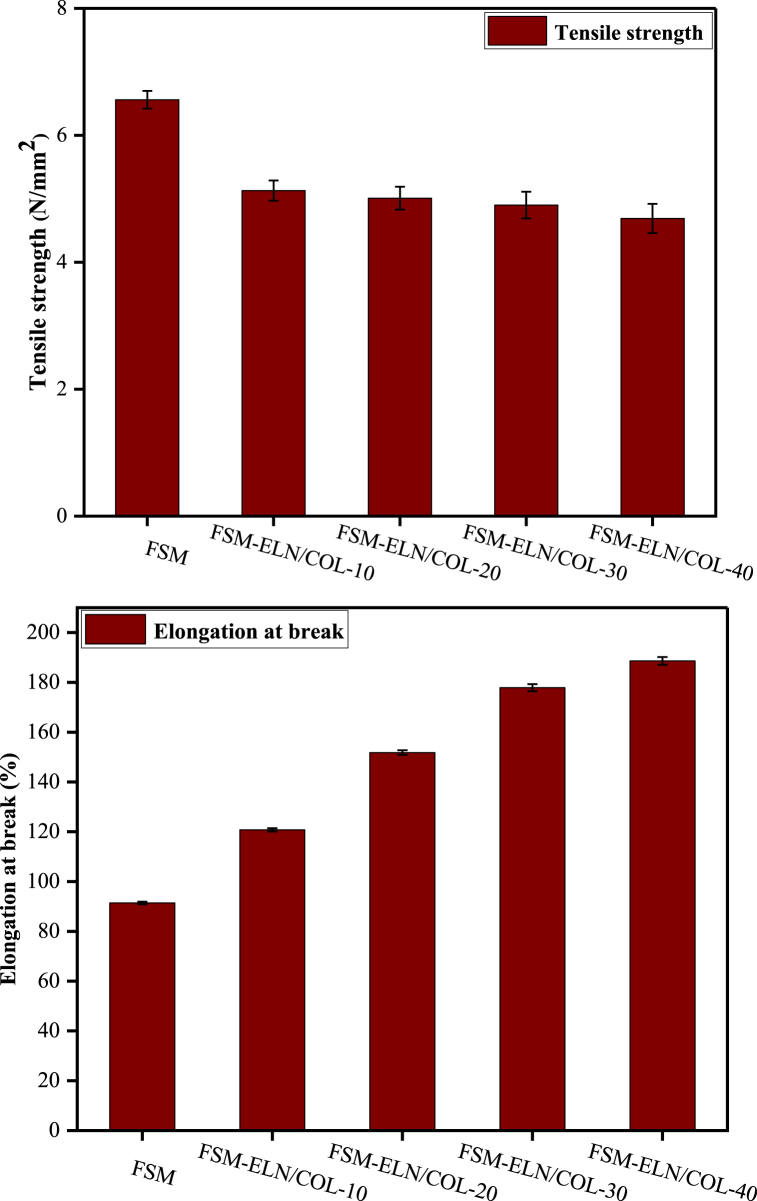
Tensile strength and elongation at break of flaxseed mucilage based biofilms with 0, 10, 20, 30 and 40 % of ELN/COL matrix.

As depicted in Fig. 6 and Table 3, the TS of biofilms with values ranging from 4.69 to 6.56 MPa. The highest tensile strength value was found for the biofilm without the addition of ELN/COL (6.56 MPa), while the lowest was found at the highest ELN/COL loading (4.69 MPa). The results showed that as addition of elastin/collagen increased, the tensile strength decrease. This might be due the fact that the addition of elastin/collagen to the flaxseed mucilage biofilm can result in a decrease in the intermolecular forces of the base matrix, in which the protein is acting as a plasticizing agent. However, the findings are better than the alginate/whey protein films prepared by S. Bishnoi et al. [49], with tensile strength of 2.0 MPa, and the wheat gluten protein/apple pectin films had a tensile strength of 1.57 MPa [58]. However, the results were significantly lower than the gelatin/cress seed gum/chitosan biofilms [59] and chia mucilage/gelatin biofilms [57], which had tensile strengths of about 7.79 and 6.97 MPa, respectively. Most importantly, the results obtained in this study meet the minimum standard for tensile strength values of edible films which is based on the Japanese industrial standard (0.3923 MPa) [60,61].

**Table 3:** Mechanical properties of FSM-ELN/COL biofilms.

Films	TS (MPa)	EAB (%)	Improvement in EAB as compare to FSM (%)
FSM	6.56 ± 0.14	91.4 ± 0.51	–
FSM-ELN/COL-10	5.13 ± 0.16	120.8 ± 0.65	32.2
FSM-ELN/COL-20	5.01 ± 0.18	151.8 ± 0.92	66.1
FSM-ELN/COL-30	4.90 ± 0.21	177.9 ± 1.39	94.6
FSM-ELN/COL-40	4.69 ± 0.23	188.6 ± 1.57	106.3

When the concentration of ELN/COL was increased from 0 to 40 % w/w, the EAB values of the biofilms increased from 91.4 % to 188.6 % as shown in Table 3 and Fig. 6. This may be due to the enhancement in chain mobility as ELN/COL matrix introduced into the FSM can increase intermolecular spacing by reducing intermolecular and intramolecular chain interaction. In addition, due to the elastin present in the protein matrix**,** the elongation of biofilms was improved. The elongation of the edible film according to the Japanese Industrial Standard (JIS) is minimal at 70 % [60]. In this study, the lowest edible biofilm elongation is 91.4 %, which means that it does meet the JIS standard. Similar results have been reported by other researchers for protein/polysaccharide-based biofilms [49].

#### 3.3.6 FTIR spectra of biofilms

**Fig. 7:**
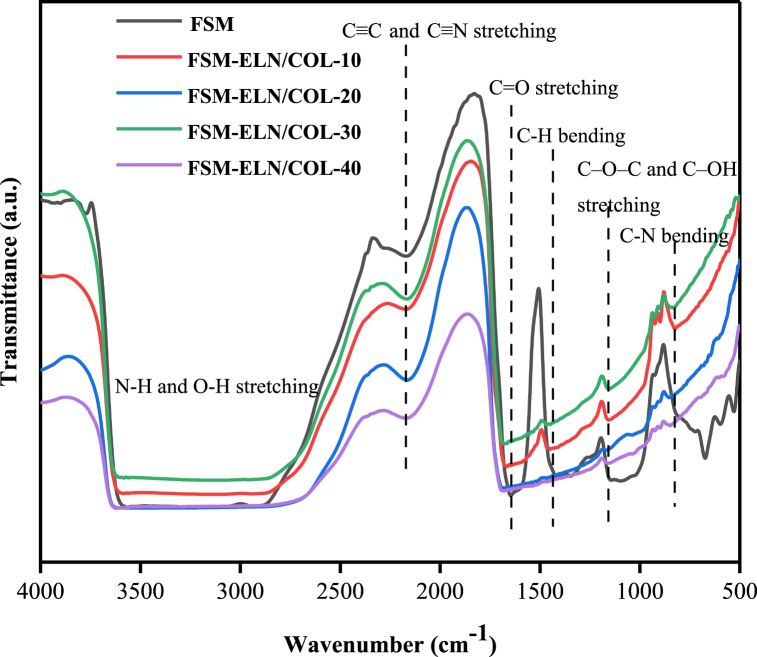
FTIR spectra of FSM-ELN/COL biofilms.

The FT-IR spectra of FSM-ELN/COL biofilms were analyzed to study the chemical interaction between FSM and ELN/COL. It can be seen from the spectrum in Fig. 7, the FSM-ELN/COL biofilms coincided with broadening bands between 3500 and 3200 cm^−1^ ascribed to the –OH and symmetric and asymmetric stretching of –NH bonds in the FSM and ELN/COL structures [62]. Moreover, as the content of ELN/COL increased from 0 % to 40 %, the C=O and N–H stretching of amide I, amide II, and amide III regions of the FSM-ELN/COL biofilms changed significantly. The changes in the position and peak intensity of these bands indicated that the hydroxyl in FSM interacted with the amino groups in ELN/COL biofilms to form hydrogen bonds, which had good compatibility [63,64]. More interestingly, a new peak at 830 cm^−1^ appeared in FSM-ELN/COL biofilms, which is related to the C–N out-of-plane wagging of amide I of ELN/COL [65,66]. The above results proved that ELN/COL had been successfully dispersed in the FSM matrix.

#### 3.3.7 Thermal stability of biofilms

**Fig. 8:**
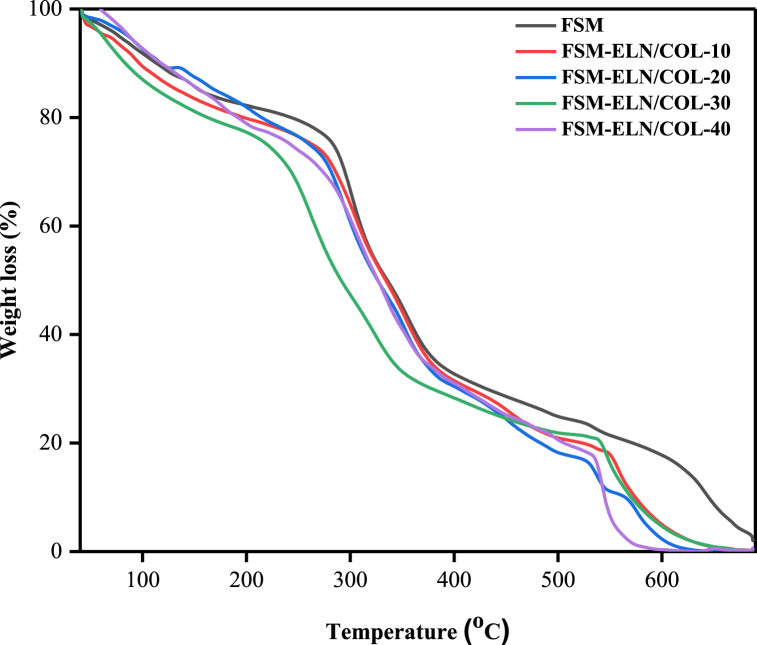
TGA curve of FSM-ELN/COL biofilms.

The thermogravimetric analysis (TGA) curves are presented in Fig. 8 and further used to analyze the thermal stability of the biofilms (Table 4). The weight loss occurred in three main thermal degradation stages. The initial degradation stage was observed between 40 and 200 °C with weight loss ranging from 10.34 to 15.06 %. The weight loss at this stage could be linked with the dehydration and vaporization of low molecular weight components such as unreacted glycerol and loosely bound water, which are available in biofilms [67,68]. The second degradation stage was revealed between 240 and 400 °C with weight loss ranging from 42.43 to 47.45 %, which attributes to the thermal decomposition of main polymeric chains [69,70]. In the final stage, which is above 500 °C can be associated with complete oxidation of polymer chains [71]. All TGA scans showed similar decomposition curves for all the biofilms, which indicates good molecular miscibility and compatibility between FSM and ELN/COL.

**Table 4:** Weight loss related to each stage of TGA curve and T_max_ from DTG of FSM-ELN/COL biofilms.

Biofilms	Weight loss (%)			Residue (%)	DTG (T_max,_^o^C)
	First stage	Second stage	Third stage		
FSM	19.02	47.41	9.06	24.51	301
FSM-ELN/COL-10	21.66	45.76	17.15	15.43	305
FSM-ELN/COL-20	21.89	43.27	18.14	16.70	295
FSM-ELN/COL-30	20.92	46.06	13.54	19.48	287
FSM-ELN/COL-40	23.10	42.72	15.03	19.15	304

**Fig. 9:**
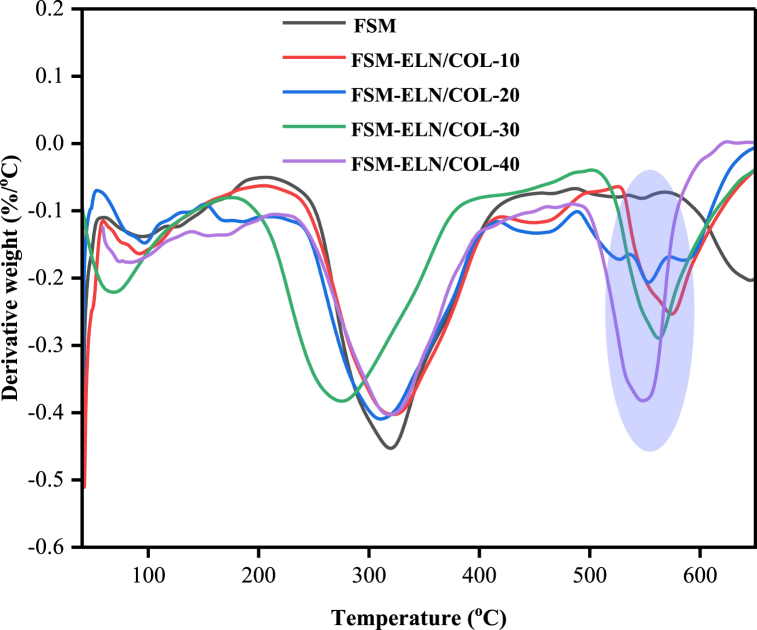
DTG curve of FSM-ELN/COL biofilms.

The major thermal decomposition of biofilms showed different trends, as depicted in DTG thermograms (Fig. 9). All the FSM-based biofilms obtained the major thermal decomposition at a range of 287 °C–305 °C. Sample FSM-ELN/COL-10 showed the higher thermal stability (305 °C), while samples with 30 % w/w of ELN/COL showed decomposition around 287 °C. For ELN/COL dispersed FSM-based biofilms decomposition was shown at 538 °C–558 °C due to the decomposition peak of the high molecular weight protein fraction of the ELN/COL matrix. These results suggest that less energy is needed to initiate its degradation process with a loading rate of ELN/COL matrix. The addition of ELN/COL to the FSM matrix shifted the thermal decomposition of FSM-ELN/COL blend biofilms to a lower temperature (Fig. 9). This decreasing effect on the thermal stability of the FSM-based biofilms might be explained by the fact that the introduction of the ELN/COL into FSM created loosely intertwined FSM chains that had higher thermal stability [70]. Other studies with comparable findings have been published [16,23]. The blend system has one distinct maximum decomposition rate peak, which explains the preferred compatibility between the ELN/COL and FSM (see Fig. 9).

#### 3.3.8 Differential scanning calorimetry (DSC)

**Fig. 10:**
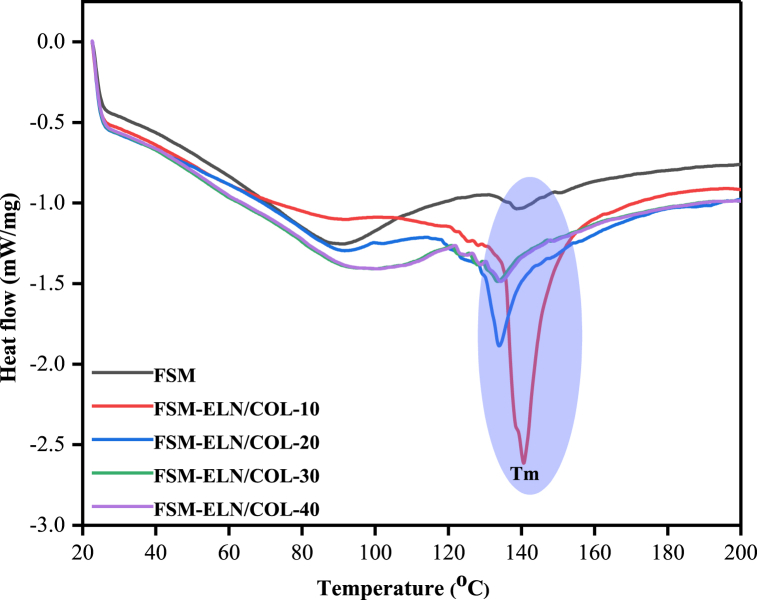
DSC thermograms of FSM-ELN/COL biofilms.

The influence of protein interaction with polysaccharides on the thermal properties of biofilms range taken for thermal scanning was 40–200 °C on a rate of 10 °C/min at N_2_ atmosphere. As shown in Fig. 10 a single Tg was observed in all biofilms, indicating good compatibility of FSM with the ELN/COL matrix [72]. The addition of ELN/COL reduced the Tg of the biofilms compared with FSM film. It shows the formation of hydrogen interactions and the increment of moisture contents [73]. Moreover, the biofilms showed Tm values in the range of 134 °C to 140 °C with ELN/COL presence decreasing the Tm position upon interaction with FSM.

#### 3.3.9 X-ray diffraction (XRD) analysis

**Fig. 11:**
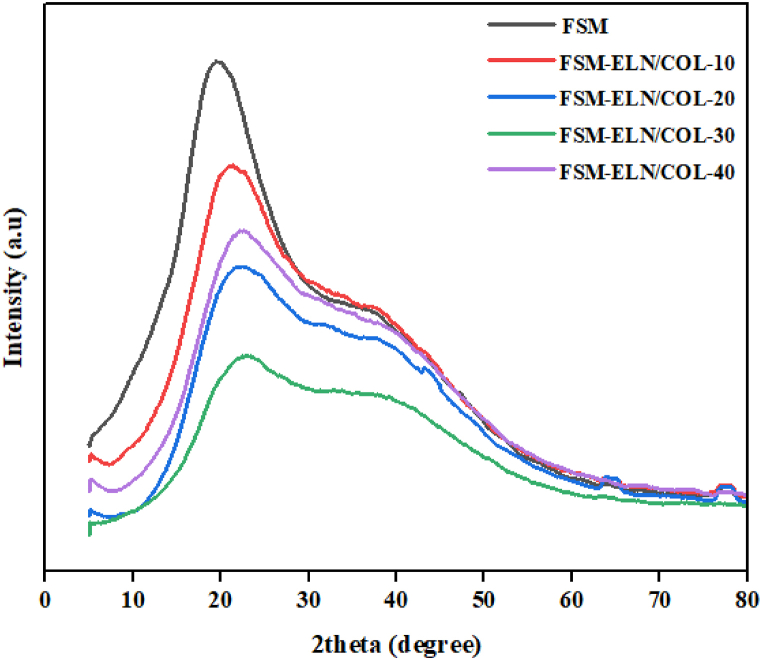
XRD pattern of FSM-ELN/COL biofilms.

XRD analysis was carried out to monitor a possible change of crystallinity of obtained biofilms and assess the compatibility of different components. As shown in Fig. 11, the XRD pattern of pure FSM displays one main diffraction peaks at 2θ = 20°, characteristic of an amorphous phase [74].

Fig. 11 showed the crystal structure of the biofilm was slightly changed when ELN/COL was blended with FSM; comparing with pure FSM biofilm, the crystalline peak at 2θ = 20° became broad with reduced intensity. This phenomenon illustrated that the addition of ELN/COL cause slight change on the conformation of FSM and decreased the regular arrangement of the FSM chains. This may be due to intermolecular interaction between the hydroxyl group of the FSM and amine groups of ELN/COL, which limited the movement of molecules and thus prohibited crystallization [75]. Overall, there was no newly sharp peak observed over the range of 2θ degree in blend biofilms suggesting that there was good compatibility and interaction among different components in the biofilms [76].

#### 3.3.10 Image analysis for dispersion of elastin/collagen matrix into FSM

**Fig. 12:**
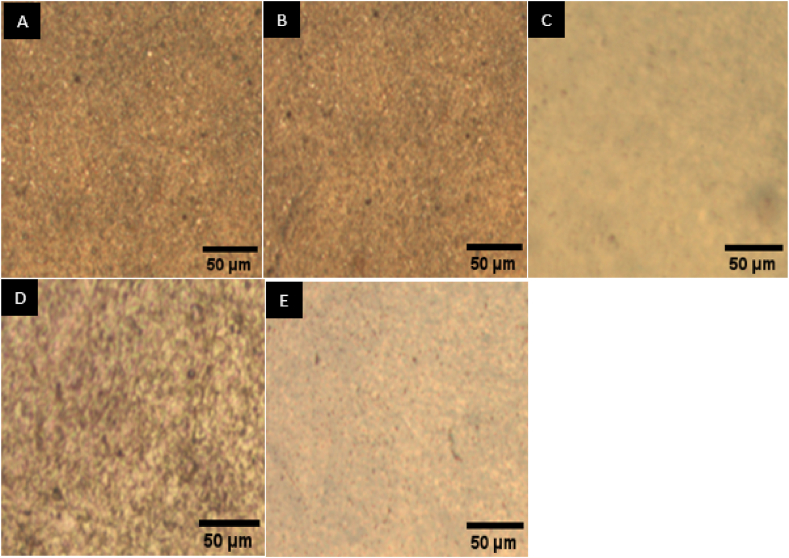
Optical microscope analysis (A) FSM film, (B) FSM-ELN/COL-10 (C) FSM-ELN/COL-20, (D) FSM-ELN/COL-30 and (E) FSM-ELN/COL-40 biofilms.

The optical microscope image analysis was used to study the dispersion of the elastin/collagen matrix into the flaxseed mucilage. The investigation was done for the FSM/ELN/COL biofilm matrix with loading rates ranging from 0 % to 40 % w/w. Biofilms exhibit smooth and homogeneous surfaces, as shown in Fig. 12. The biofilms were prepared by suspension of elastin/collagen into the flaxseed mucilage solution and drying. It indicates the elastin/collagen matrix remained stable during the film forming process.

## 4 Conclusion

Flaxseed mucilage based biofilms with 0 %–40 % w/w loading rate of elastin/collagen matrix was developed. The physicochemical properties, FT-IR analysis, mechanical and thermal properties of the biofilm were investigated. The results of this study suggested that incorporation of elastin/collagen (ELN/COL) into flaxseed mucilage (FSM) has a considerable influence on physicochemical properties of the obtained FSM-ELN/COL biofilms. The incorporation of the ELN/COL provided the blends greater elongation and also resulted in intermolecular interaction, which was confirmed by ATR-FTIR spectra. It was demonstrated through TGA that the flaxseed mucilage used alone resulted in biofilms with greater thermal stability. With the addition of ELN/COL, the thickness, solubility and opacity of flaxseed mucilage biofilm were significantly increased, but the tensile strength was decreased. XRD diffraction shows that the intensity of the characteristic peak at 2θ = 20° of FSM decreased with increasing ELN/COL concentrations in the biofilm. These results indicate that the FSM-ELN/COL biofilms would be used as edible food packaging material.

**Table:** **W1.Proximate analysis of mucilage, starch and**p**rotein**

Proximate analysis	Test performed	Outcomes
Mucilage	Ruthenium red test	**+**
Starch	Iodine test	**-**
Protein	Biuret test	**-**

Note; + = present, − = absent.

## CRediT authorship contribution statement

**Genet Tewelde Hailu:** Writing – original draft, Validation, Methodology, Investigation, Formal analysis, Data curation, Conceptualization. **Fekadu Lemessa:** Validation, Supervision, Project administration, Methodology, Formal analysis, Conceptualization. **Melakuu Tesfaye Alemea:** Writing – review & editing, Visualization, Validation, Supervision, Resources, Project administration, Methodology, Investigation, Data curation, Conceptualization.

## Declaration of competing interest

The authors declare that they have no known competing financial interests or personal relationships that could have appeared to influence the work reported in this paper.
